# The impact of integrative psychotherapy on psychological well-being and quality of life in patients with breast cancer undergoing active treatment

**DOI:** 10.25122/jml-2024-0375

**Published:** 2025-02

**Authors:** Elena Gabriela Vâlcu, Silvia Fotea, Anamaria Ciubară, Laura Florentina Rebegea

**Affiliations:** 1 Medical Clinical Department, Dunarea de Jos University, Galati, Romania

**Keywords:** breast cancer, integrative psychotherapy, depression, EORTC QLQ-C30, EORTC QLQ-C30, European Organisation for Research and Treatment of Cancer Core Quality of Life Questionnaire, QLQ-BR23, Breast Cancer-Specific Module of the EORTC Quality of Life Questionnaire, BDI-II, Beck Depression Inventory-II, ST, Systemic Therapy Side Effects – Symptoms related to systemic cancer treatment, HL, Upset by Hair Loss – Emotional distress caused by hair loss, AS, Arm Symptoms – Pain, swelling, or mobility issues in the arm, BS, Breast Symptoms – Pain or discomfort in the affected breast, BI, Body Image – Perception of physical appearance and body changes, FP, Future Perspective – Patient’s outlook on future health and well-being, SF, Sexual Functioning – Impact of breast cancer on sexual activity and function, SE, Sexual Enjoyment – Satisfaction and pleasure derived from sexual activity

## Abstract

This study aimed to evaluate the effects of individual integrative psychotherapy on patients with breast cancer undergoing active treatment. A total of 23 patients diagnosed with breast cancer and treated at the Oncology Department of Sf. Ap. Andrei Clinical Emergency County Hospital, Galati, Romania, participated in the study. The psychotherapy sessions were conducted weekly for each patient, and family counseling sessions were held twice a month over 6 months. Three standardized psychological instruments were administered before and after therapy to evaluate the effectiveness of the psychotherapeutic intervention. The European Organisation for Research and Treatment of Cancer (EORTC) Quality of Life Questionnaire-BR23 (QLQ-BR23) was used to assess disease-specific quality of life. The Beck Depression Inventory-II (BDI-II) measured the severity of depressive symptoms, while the 16 Personality Factors Questionnaire (16PF) evaluated personality traits related to coping and emotional resilience. A comparative analysis of pre-and post-therapy results revealed significant improvements across multiple domains. Participants reported an overall improvement in quality of life, with reduced breast and arm pain. Functional well-being improved, particularly in the domains of sexuality and future perspective. The study also examined the psychological impact of different treatment modalities, including surgery and chemotherapy, and their influence on emotional adaptation. Postoperative changes in quality of life were closely monitored, allowing for a comprehensive evaluation of the benefits of psychotherapy in alleviating the emotional and physical burden associated with breast cancer treatment.

## INTRODUCTION

Breast cancer stands as a main cause of morbidity and mortality among women around the world [1]. In Romania, approximately 12,000 new cases are diagnosed annually, according to Globocan [2]. Despite the implementation of dedicated screening, education, and prevention programs, substantial disparities remain compared to other European Union countries in terms of early detection and treatment outcomes [3]. The evaluation of cancer treatments has focused on clinical endpoints, such as the rate of survival, remission rate, and physical health indicators [1]. However, in recent years, there has been a growing recognition of the psychosocial and emotional impact of breast cancer, highlighting the need to assess quality of life (QoL) as a key outcome. Psychological distress, depression, and impaired social integration are common in patients with breast cancer, particularly during active treatment phases. These factors are closely linked to each patient’s subjective illness experience, influencing treatment adherence and overall prognosis. Evaluating patients’ quality of life is very important for psychologists involved in oncological studies [4]. Given the multidimensional nature of cancer care, a holistic approach is necessary, integrating psychological, cognitive, and emotional support alongside medical treatment [5]. An integrative therapeutic approach—which considers psychological, emotional, and behavioral aspects—can significantly improve psychological resilience, body image, and overall quality of life in patients with breast cancer.

## MATERIAL AND METHODS

### Study design and participants

This study employed a prospective observational design to evaluate the effects of individual integrative psychotherapy on breast cancer patients undergoing active treatment. The study included 23 female patients aged 35–55 years who were diagnosed with non-metastatic breast cancer and treated at the Oncology Department of Sf. Ap. Andrei Clinical Emergency County Hospital, Galati, Romania.

Participants were selected based on the following inclusion criteria: (1) confirmed diagnosis of non-metastatic breast cancer, (2) undergoing active treatment, including surgery and/or chemotherapy, (3) absence of major psychiatric disorders that could interfere with psychological assessment, and (4) willingness to participate in a psychotherapy intervention program. All participants had at least secondary or higher education, were married, and had minor or adult children. The study ensured homogeneity by selecting participants with similar pathology and treatment phases.

### Psychotherapeutic intervention

The intervention consisted of a structured integrative psychotherapy program designed to address psychological distress, improve emotional adaptation, and enhance coping strategies. Each patient received weekly individual psychotherapy sessions, while family counseling was provided twice a month. The total duration of the intervention was 6 months. The therapy was based on an integrative approach that combined cognitive-behavioral techniques, emotional processing strategies, and resilience-building exercises. The sessions aimed to help patients navigate the psychological challenges associated with their cancer diagnosis and treatment, with a focus on improving emotional well-being, self-perception, and overall quality of life.

### The supportive therapeutic relationship

A supportive therapeutic relationship is very important in developing the therapeutic process facilitating emotional security, trust, and engagement [6]. Psychotherapists devoted significant time to understanding the patient’s life, family dynamics, and personal experiences to develop an individualized approach. By showing a genuine interest in the patient’s life, the therapist demonstrated empathy and understanding of their unique reality. This approach fostered a sense of emotional involvement, helping the patient develop trust in the therapeutic process. This trust encouraged them to confront avoidance behaviors and explore emotionally charged experiences [7]. Through guided discovery, the psychologist and patient could identify relevant patterns in how challenges were structured, allowing for a deeper understanding of the recurrent difficulties in various aspects of their life [8]. Additionally, discussing personal values and life experiences revealed important strengths and positive relationships that served as psychological resources during treatment. The supportive therapeutic relationship, the guided discovery, and the integrative approach are key elements of an effective psycho-oncological intervention [9].

The challenge in working with patients with cancer involved maintaining a balance between simply being present and actively facilitating change. The therapeutic process required a careful interplay between acceptance and transformation, emotional exploration and problem-solving, to support the patient's psychological adjustment to the illness [10].

The counseling process incorporated an exploration of personal values, relationships, and meaningful activities to help patients reconnect with aspects of their lives that gave them purpose. Patients were asked reflective questions such as:

- Who is important in your life?
- What was important to you before your diagnosis?
- What would you do/not do if you felt better/went into remission?
- What do you miss most when being in the hospital?
- What kind of role model do you wish to be for those around you?
- What kind of presence do you want to be?

The exploration also extended to identifying the three most important relationships in the patient’s life and the activities she enjoyed the most. By engaging in these reflections, patients could integrate aspects of their identity and values into their coping strategies, reinforcing resilience and psychological well-being.

### Intervention plan

The psychotherapeutic intervention focused on the depression component, as these were the predominant psychological difficulties experienced by the study participants. Integrative therapy was implemented, with each patient receiving one session per week. Beck's cognitive triad was central to the approach, given the common implicit belief among participants that “cancer = death" [11]. This belief can significantly impact emotional processing and lead to psychological distress, affecting survival instincts, sexual functioning, nutritional habits, sleep, and the ability to experience pleasure. A key component of the intervention was identifying and addressing maladaptive cognitive patterns. Through structured therapeutic dialogue, patients were encouraged to recognize automated negative thoughts, cognitive distortions, and prejudicial beliefs related to their diagnosis. The therapeutic process involved restructuring these distorted thoughts and introducing alternative, adaptive perspectives. Personalized therapeutic contracts were formulated, including structured exercises aimed at breaking depressive cycles and reinforcing emotional resilience.

### Psychotherapeutic approach and techniques

The initial psychological evaluation suggested, consistent with prior research that many patients exhibited dysthymic traits in their premorbid personality [8]. In cases of more severe psychological distress, this manifested as adjustment disorder with depressive mood, as classified in the Diagnostic and Statistical Manual of Mental Disorders, Fifth Edition (DSM-5) [12]. Based on this assessment, the intervention was designed to systematically address maladaptive cognitive and emotional responses through a structured therapeutic approach. The intervention plan was structured to achieve the following goals:

1. Strengthen adaptive mechanisms to cope with cancer diagnosis and treatment.
2. Prevent emotional breakdowns by developing resilience-building strategies.
3. Reduce the fear of relapse through cognitive and emotional processing.
4. Conduct psychological re-evaluation of emotional states, including objective assessment of progress.

The intervention included individualized strategies targeting dysfunctional cognitive and behavioral processes. Several established psychotherapeutic approaches were integrated:

**Cognitive-behavioral therapy (CBT)**: patients were guided to identify cognitive distortions, irrational beliefs, and automatic negative thoughts. Therapeutic exercises helped them challenge these distortions and replace them with more balanced perspectives.

**Transactional analysis (TA)** [13]: life scripts, internalized drivers, and injunctions were explored to identify deep-seated patterns influencing emotional responses.

**Simonton method** [14]: patients engaged in guided imagery, relaxation exercises, and cognitive reframing techniques to manage stress, fears of relapse, and existential concerns related to illness progression.

### Family counseling and emotional processing

Recognizing the broader impact of breast cancer on family dynamics, family counseling sessions were included to support the emotional well-being of both patients and their loved ones. Family members often expressed feelings of helplessness, grief, or frustration regarding the patient’s illness and treatment. Counseling sessions provided a structured space to address these concerns, offering guidance on effective communication, emotional support, and coping strategies. In many cases, family members exhibited anger directed toward healthcare providers due to perceived delays in diagnosis or concerns about treatment efficacy. Others experienced suppressed grief, struggling with feelings of guilt over the patient’s condition. The therapy sessions helped address these issues, fostering a more supportive family environment and encouraging open dialogue. Patients described the therapy sessions as a safe space to discuss personal struggles, including body image concerns, the psychological impact of mastectomy, and feelings of diminished femininity. These discussions were essential in helping patients regain self-confidence and navigate the emotional challenges of living with breast cancer. All these interventions allowed a readjustment and a redefinition of the entire family system.

### Psychological assessment tools

To evaluate the effectiveness of the psychotherapeutic intervention, standardized psychological instruments were administered before and after therapy. The effectiveness of the intervention was assessed at multiple time points: baseline (t = 0), 1 month (t = 1), 3 months (t = 3), and 6 months (t = 6). The European Organization for Research and Treatment of Cancer Quality of Life Questionnaire-BR23 (EORTC QLQ-BR23) was used to measure disease-specific quality of life. This validated instrument assesses functional well-being (e.g., body image, future perspective, sexual functioning, and sexual enjoyment) and symptom-related domains (e.g., systemic therapy side effects, hair loss distress, arm, and breast symptoms). Higher scores on functional scales indicate better well-being, whereas higher scores on symptom scales indicate greater symptom severity [15]. The Beck Depression Inventory-II (BDI-II) was used to assess the severity of depressive symptoms, providing insight into the emotional burden experienced by the patients [16]. The 16 Personality Factors Questionnaire (16PF) was also administered to evaluate personality traits, coping mechanisms, and emotional resilience [17].

The Breast Cancer-Specific Module (BR-23) of the EORTC QLQ-C30 was also utilized as an additional measure. This 23-item Likert-scale questionnaire was designed specifically to assess the quality of life in patients with breast cancer, capturing both the physical and emotional challenges associated with the disease and its treatment. The QLQ-BR23 incorporates two domains of quality of life: (1) the symptom domain includes systemic therapy side effects (ST), hair loss distress (HL), arm-related symptoms (AS), and breast-related symptoms (BS), and (2) the functional domain, which evaluates body image (BI), future perspective (FP), sexual functioning (SF), and sexual enjoyment (SEE). Each scale and item is scored in a range of 0 to 100. Higher scores on functional scales reflect a higher level of well-being, while higher scores on symptom scales indicate greater symptom burden.

### Statistical analysis

All statistical analyses were conducted using MedCalc software to determine the efficacy of the psychotherapeutic intervention in improving symptom burden and functional well-being in patients with breast cancer. Given that the study involved repeated measurements on the same participants before and after therapy, a paired samples *t*-test was applied to compare pre-and post-intervention scores across multiple psychological and quality-of-life variables.

The statistical hypotheses were defined as follows:

*The null hypothesis (H_0_):* There is no significant difference between the pre-and post-therapy measurements, indicating that the intervention had no measurable effect.

*The alternative hypothesis (H_1_):* There is a significant difference between the pre-and post-therapy measurements, demonstrating that the intervention had a statistically significant impact on psychological and quality-of-life outcomes. Student’s *t*-test was used to compute the mean difference for each outcome variable, along with the associated 95% confidence interval (CI) and two-tailed *P* values. A *P* value threshold of 0.05 was set for statistical significance, meaning that values below this level indicated a clinically meaningful effect of the intervention. The analysis was performed on the following variables: (1) symptom scales: systemic therapy side effects (ST), arm symptoms (AS), breast symptoms (BS), and upset by hair loss (HL), and (2) Functional scales: body image (BI), future perspective (FU), sexual functioning (SEF), and sexual enjoyment (SEE). The results of the statistical analysis, including *P* values, are detailed in Table 2, while Figures 3–10 visually represent the observed changes..

**Figure 1:**
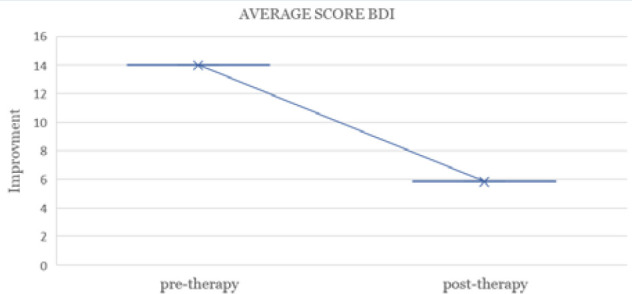
Reduction in average BDI scores pre- and post-therapy

**Figure 2:**
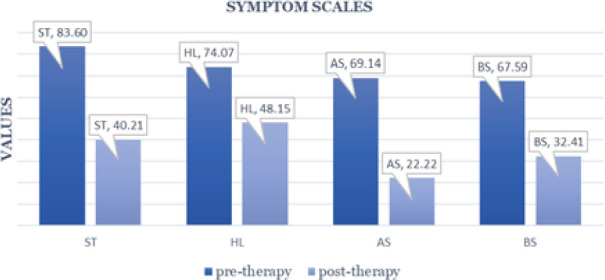
Symptom scale – EORTC Questionnaire QLQ-BR23

**Figure 3:**
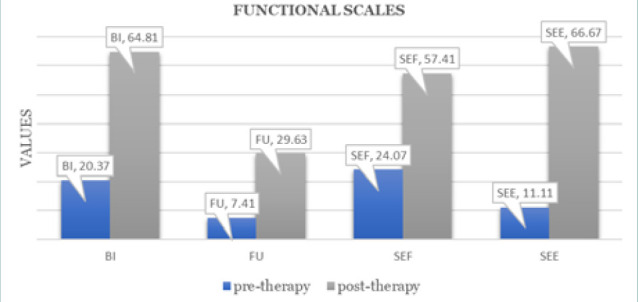
Activity scale - EORTC questionnaire QLQ-BR23

**Figure 4:**
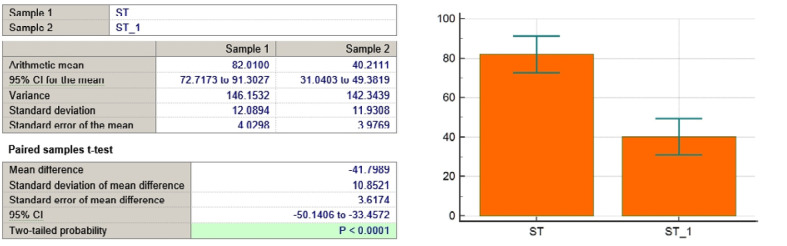
ST variable

**Figure 5:**
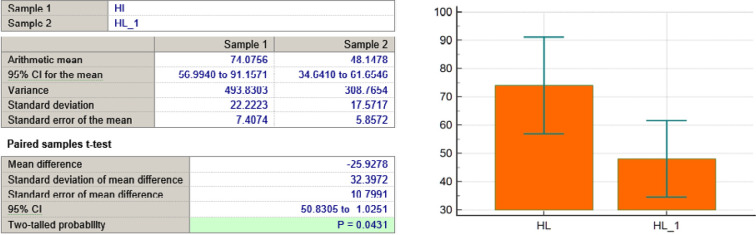
HL variable

**Figure 6:**
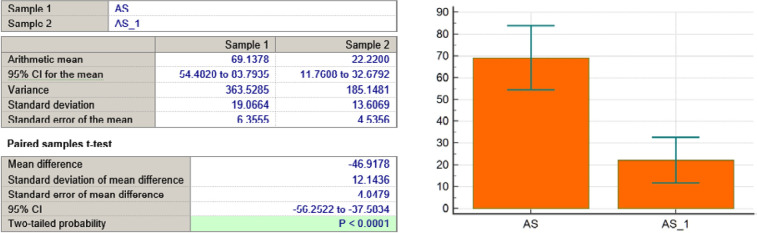
AS variable

**Figure 7:**
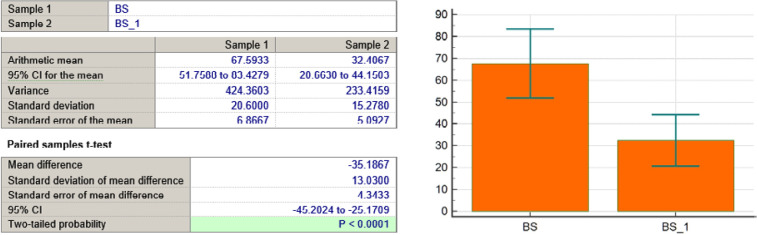
BS variable

**Figure 8:**
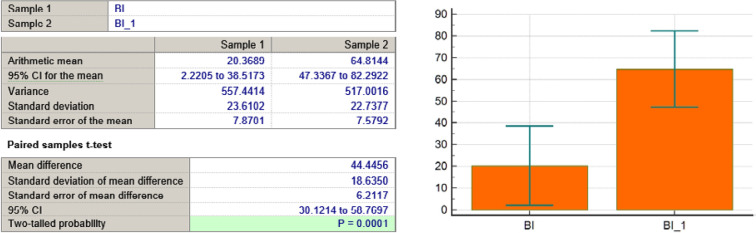
BI variable

**Figure 9:**
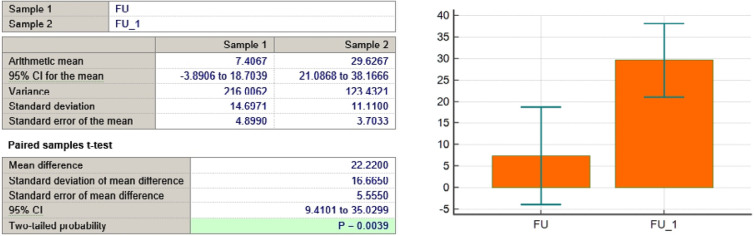
FU variable

**Figure 10:**
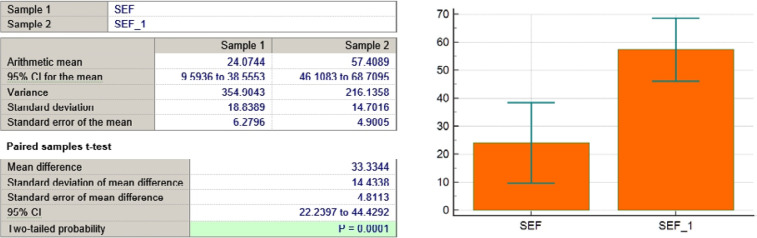
SEF variable

**Figure 11:**
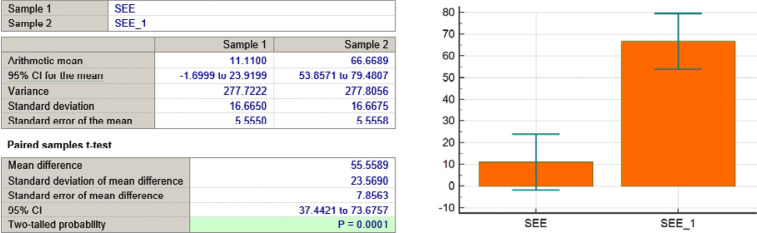
SEE variable

## RESULTS

### Patient history and psychological profiles

The analysis revealed a high prevalence of significant life stressors among participants. It was found that 75% of participants reported experiencing at least one major bereavement or personal loss within the past 5 years, while 60% had similar experiences in the last 2 years.

### Personality traits and coping styles (16PF results) before and after intervention

The 16PF was administered to evaluate personality traits relevant to coping mechanisms and emotional resilience before and after the intervention. Baseline results indicated a pattern of emotional detachment, social withdrawal, and anxiety-prone tendencies. The findings are summarized in Table 1. 93% of patients exhibited depressive tendencies (Factor F score <6), and 91% showed signs of generalized anxiety (Factor Q4 score >5). Post-intervention analysis showed significant improvements in key psychological factors. Factor A (Schizothymia-Cyclothymia), Factor L (Confident Attitude-Suspicion), and Factor Q4 (Absence of Anxiety-Anxiety), which are categorized as feeling structures, had significant positive changes. Additionally, Factor C (Ego Strength) improved significantly. A comparative analysis between pre- and post-therapy scores revealed a >12% increase in Factor A and Factor C, suggesting greater relational availability and emotional stability. Factor L decreased by 15%, indicating a reduction in interpersonal suspicion, while Factor Q4 decreased by 20%, reflecting a reduction in generalized anxiety levels.

**Table 1:** Baseline 16PF results in breast cancer patients

Personality Factor	Description	% of patients	Interpretation
Factor A (Schizothymia-Cyclothymia)	Cold, rigid, distant vs. warm, sociable	17%	Emotional detachment, low affectivity
Factor B (Global Intelligence)	Cognitive ability	88%	Average intelligence
Factor C (Ego Strength)	Emotional stability vs. lability	85% (score <5)	Emotional lability, low frustration tolerance
Factor E (Subordination-Dominance)	Passive vs. assertive	75% (score 4-8)	Latent/manifest aggression
Factor F (Depression-Sociability)	Cheerful vs. withdrawn	93% (score <6)	Depression, social isolation tendencies
Factor G (Super-Ego Strength)	Rule-conscious vs. impulsive	93% (score 4-10)	Strong guilt tendencies
Factor H (Boldness-Shyness)	Outgoing vs. introverted	55% (score <5)	Introversion, privacy-seeking behavior
Factor I (Sensitivity-Rationality)	Emotional vs. logical	63% (score <6)	Emotional repression
Factor L (Trust-Distrust)	Trusting vs. suspicious	81% (score 5-9)	Increased suspicion
Factor M (Abstraction-Practicality)	Imaginative vs. realistic	51% (score 1-5)	Rigid, inflexible thinking
Factor N (Naivety-Cunning)	Straightforward vs. strategic	90% (score 5-10)	Social caution, emotional detachment
Factor O (Security-Guilt)	Confident vs. apprehensive	85% (score 5-10)	Apprehension, guilt tendencies
Factor Q1 (Conservatism-Radicalism)	Traditional vs. experimental	74% (score 5-9)	Oppositionist tendencies
Factor Q2 (Dependence-Autonomy)	Dependent vs. independent	66% (score 5-9)	Balanced autonomy
Factor Q3 (Anxiety Control)	Emotionally regulated vs. unstable	90% (score 4-10)	Moderate control over anxiety
Factor Q4 (Generalized Anxiety)	Relaxed vs. tense	91% (score 5-10)	Generalized anxiety, low criticism tolerance

### Impact of psychotherapy on psychological well-being

At one-month post-intervention (t = 1), there was a temporary increase in depressive symptoms (BDI-II mea *n* = 10.67), which appeared to be a paradoxical effect of the therapeutic process. This initial increase was interpreted as an activation of underlying grief and emotional distress, consistent with the process of psychological confrontation with illness-related experiences. However, by 6 months (t = 6), depressive symptoms had significantly decreased, correlating with improved emotional well-being and quality of life (Figure 1).

As shown in Figure 2, there was a significant reduction in symptom severity by the end of psychological therapy. The most notable improvements were observed in arm pain (decreasing from 69% to 22%) and breast pain (from 68% to 32%).

In addition to symptom reduction, functional well-being significantly improved across multiple domains, particularly in sexual function and future perspective. Figure 3 illustrates that sexual enjoyment increased from 11% to 67%, and future perspective improved from 7% to 30%, demonstrating an enhanced sense of self-perception and optimism. Table 2 summarizes the trends in symptom reduction and functional scale improvements based on the EORTC QLQ-BR23 assessment.

**Table 2:** Symptom and activity scale trends at the end of the psychotherapy programs

Symptom scales (%)				Functional scales (%)			
ST	HL	AS	BS	BI	FU	SEF	SEE
51.90	35.00	67.86	52.06	218.18	300.00	138.46	500.06

At the end of the six-month psychotherapy program, substantial improvements were observed in both symptom relief and functional well-being. In percentage terms, pain in the arm and breast decreased by 68% and 52%, respectively, along with a comparable reduction in the side effects of systemic therapy. In the areas of functionality, there is a significant increase in the Sexual Enjoyment area by over 500% and the Future Perspective area by 300%.

As seen in Table 3 and Figures 4-11, *P* values were lower than the limit value of 0.05. Therefore, there were significant statistical differences between the values before and after the treatment, suggesting that the treatment had a determining influence on the patient’s life.

**Table 3:** Statistical significance of symptom and functional scale improvements

Symptom scales				Functional scales			
ST	HL	AS	BS	BI	FU	SEF	SEE
*P* < 0,0001	*P* = 0,0431	*P* < 0,0001	*P* < 0,0001	*P* = 0,0001	*P* = 0,039	*P* = 0,0001	*P* = 0,0001

## DISCUSSION

The findings of this study highlight the significant impact of integrative psychotherapy on the psychological well-being and quality of life of breast cancer patients undergoing active treatment. The results demonstrate that psychological interventions are a crucial component of oncological care, complementing medical and surgical treatments. The statistical analyses confirmed that therapy played a determining role, with significant improvements in depression levels, emotional resilience, and quality-of-life indicators.

Breast cancer treatment often involves radical interventions such as mastectomy and chemotherapy, both of which can lead to severe psychological distress due to their physical and symbolic implications. The loss of a breast, in particular, affects self-perception, femininity, and body image, contributing to increased vulnerability, depression, anxiety, and social withdrawal. The results indicate that patients in this study experienced improvements in body image perception, which is essential for psychological recovery. However, certain aspects, such as distress related to hair loss, showed less significant improvements, suggesting that further targeted interventions may be needed to address this issue. The goal of psychotherapy is to restore the self-image, and, at the same time, it aims to return the patient to her family and social cluster [18].

Analysis of the participants’ psychological responses revealed experiences of loss, breakups, and trauma, often expressed through contradictory communication styles. Patients exhibited emotional detachment and low engagement when discussing painful life events. The collected data confirm the observations of Leshan and Worthington [19] and in the later work of Schmale and Iker [20], who emphasized the importance of the loss of a significant relationship in the pathogenesis of the neoplasm.

The personality structure of the patients in this study appeared to be closely related to the functioning styles of their families of origin. Participants frequently reported intra-familial relationships characterized by low intimacy and significant emotional distance from parental figures. These dynamics align with previous studies suggesting that childhood relational patterns contribute to coping mechanisms in adulthood [13]. Many patients exhibited life scripts such as ‘be perfect', ‘be strong', and ‘please others', which are associated with a heightened sense of self-expectation, emotional suppression, and a reluctance to express vulnerability. These deep-seated cognitive and emotional patterns appear to have influenced the way participants adapted to their breast cancer diagnosis and treatment.

These signals were interpreted as tendencies to submit, to receive consensus and approval. The participants in the study were characterized by a weak manifestation of affect and emotions, tendencies to suppress needs, and an attempt to adapt, even in unpleasant situations, to receive approval and acceptance from others. A significant concern among participants was the impact of surgical interventions on body image and self-esteem. The experience of hair loss, mastectomy, or physical changes led to heightened distress, with many patients expressing feelings of shame, alienation, and body disharmony. Such an organization of the psychological response justifies paying attention to the patient's mental component as an important point in the therapeutic approach.

A psychological discomfort characterized by anxiety and depressive symptoms was frequently observed. The initial depressive reaction, which began with the experience of reduced quality of life, was further reinforced by the treatment process itself. Surgery and chemotherapy, along with their side effects, intensified feelings of body ‘disharmony,’ exacerbating emotional distress. The experience of rejection and profound alienation from one's self-image exacerbated depressive symptoms. This psychological response to a 'real wound' underscores the need for heightened attention to the patient's emotional well-being and highlights the importance of psychotherapeutic intervention in addressing these challenges.

Functional analysis (FA), developed by Will Davis, is a recent body-centered psychotherapeutic approach rooted in the humanistic framework of Carl Rogers and the research of Wilhelm Reich [21]. FA emphasizes the vital force as a creative and unifying process of both psyche and soma, manifesting through thoughts, emotions, behaviors, and physical states. FA techniques integrate gentle physical interventions (such as Points and positions, breathing exercises, and eye segment work) with verbal techniques focusing on self-experience. These methods restore the body's natural energy balance, facilitate homeostasis, and promote spontaneous self-regulation. Unlike traditional diagnostic models focusing on symptom reduction, FA provides a holistic understanding of human functioning, making it a valuable complement to conventional psychotherapy.

Given that medical treatments such as chemotherapy and radiotherapy can destabilize the body, FA offers a therapeutic framework for reconnecting patients with their physical selves. Healing in this context involves creating a space for patients to accept, listen to, and understand their bodies, particularly when experiencing pain or alienation. Psychotherapists are witnesses and facilitators, helping patients rediscover their inner vitality and resilience. In addition, they can call for the vital, creative aspects, often blocked, for a stable body capable of experiencing different emotions. A more aware body is tolerant of its limits.

The study also explored the application of Gestalt techniques, such as the ‘two-chair’ method, in which the body actively informs the dialogue by enriching it with implicit suggestions. In the individual setting, it is recommended to apply guided imagery techniques that also involve body activation. Also, the possibility of patients accessing therapy should be investigated.

## CONCLUSION

The study revealed that patients had distinct psychological responses after receiving information about their diagnosis and treatment plan. Initially, concerns about survival led to the onset of depressive symptoms and a decline in quality of life. These effects were further compounded by a pronounced deterioration in self-image following surgery. During the study, specific aspects of personality evaluated as individual negative adaptation dispositions were identified, some of which are likely to be changed by psychotherapeutic treatment. The integration of psychotherapy and family counseling proved to be a highly effective strategy in addressing the emotional and psychological burden of breast cancer. By helping patients process fears, restructure negative thought patterns, and develop healthier coping mechanisms, psychotherapy contributed to significant improvements in emotional stability, reduced anxiety, and enhanced psychological resilience. These findings support the growing recognition that cancer treatment should extend beyond medical interventions to include comprehensive mental health support as an essential component of patient care.

## Data Availability

Further data is available from the corresponding author upon reasonable request.
